# Development of an RPA-CRISPR/LbaCas12a-Lateral Flow Assay for the Visual Detection of *Chrysotila dentata* (Haptophyta)

**DOI:** 10.3390/microorganisms13092203

**Published:** 2025-09-20

**Authors:** Jiating Yu, Yun Shen, Qinfei Zhang, Xuxu Luo, Yujie Zong, Chengxu Zhou, Hailong Huang, Haibo Jiang

**Affiliations:** 1 National Engineering Research Center of Marine Biotechnology and Engineering, Ningbo University, Ningbo 315211, China; 226003112@nbu.edu.cn (J.Y.); 236003479@nbu.edu.cn (Y.S.); 236005122@nbu.edu.cn (Q.Z.); xuxuluo2501130161@outlook.com (X.L.); 216003820@nbu.edu.cn (Y.Z.); zhouchengxu@nbu.edu.cn (C.Z.); 2 Key Laboratory of Aquacultural Biotechnology, Ningbo University, Ministry of Education, Ningbo 315211, China; 3 Key Laboratory of Marine Ecosystem Dynamics, Second Institute of Oceanography, Ministry of Natural Resources, Hangzhou 310012, China

**Keywords:** harmful algal blooms, *Chrysotila dentata*, detection method, RPA-CRISPR/LbaCas12a-LFD

## Abstract

*Chrysotila dentata* (Haptophyta), a harmful algal bloom (HAB) species frequently occurring in coastal waters of China, is one with strong environmental adaptability that poses a serious threat to marine ecosystems and fisheries. Current molecular detection techniques and early warning systems for this species remain limited. To address this, we developed a rapid and highly sensitive detection method for *C. dentata*. This method integrates recombinase polymerase amplification (RPA) with CRISPR-LbaCas12a and lateral flow dipstick (LFD) technologies, enabling visual readout of results. Key parameters, including the single-stranded DNA (ssDNA) reporter concentration, reaction time, and temperature, were systematically optimized. Field water sample testing demonstrated high specificity and sensitivity, achieving a detection limit of 5 × 10^−6^ pg μL^−1^ for genomic DNA under laboratory conditions and 2.82 × 10^1^ cells mL^−1^ in simulated environmental samples. The entire detection process takes only 1 h (at a constant 39 °C), and results can be directly interpreted via LFD strips. For early warning and prevention of *C. dentata* outbreaks, this assay provides a powerful, reliable, and field-ready monitoring tool.

## 1. Introduction

*Chrysotila dentata* (Haptophyta), a typical coccolithophore, produces unique calcium carbonate coccoliths that accumulate on the seafloor after cell death, serving as important biostratigraphic marker fossils [1]. Due to their strong environmental adaptability, this species is widely distributed in oceanic waters. Studies have shown that coccolithophores, including *C. dentata*, can cause large-scale harmful algal blooms (HABs) with significant impacts on water quality and marine ecosystems [2,3]. *C. dentata*, a common HAB species in China’s coastal waters [4], was first recorded along the coast of Hebei Province in 2011 [5]. Although current research on the specific environmental impacts of *C. dentata* and its closely related species remains limited, the potential of coccolithophores for toxin production and damage to fisheries underscores the need to evaluate the ecological risks posed by *C. dentata* [6,7]. Enhanced monitoring and early warning systems are therefore critical for ecological and economic protection. However, current methods for *C. dentata* detection remain confined to whole-genome sequencing and bioinformatics analysis [8], lacking rapid, field-deployable tools suitable for on-site applications.

Currently, rapid detection of harmful algae primarily relies on molecular techniques, which mainly include the following categories: (1) non-isothermal amplification techniques, such as polymerase chain reaction (PCR) [9] and quantitative real-time PCR (qPCR) [10,11]; (2) nucleic acid hybridization techniques, including fluorescent in situ hybridization (FISH); and (3) isothermal amplification techniques [12]. Although PCR and qPCR techniques offer high specificity and sensitivity, their requirements for precision instruments and long reaction times make them unsuitable for on-site rapid detection [13]. FISH allows visual detection but is constrained by sensitivity issues related to probe design and sample processing and is applicable only to species with known sequences [14]. In contrast, isothermal amplification techniques enable nucleic acid amplification at constant temperatures without sophisticated instrumentation, rendering them particularly suitable for field detection conditions [15,16,17]. Common isothermal methods include loop-mediated isothermal amplification (LAMP) [18] and recombinase polymerase amplification (RPA) [19]. While LAMP has simple reaction conditions, it requires the design of six primer pairs, making primer design complex and prone to false-positive results [20]. By comparison, RPA utilizes three core proteins to achieve efficient nucleic acid amplification within 20 min at a constant temperature of 37 °C without complex thermal cycling equipment and only requires a single pair of simple primers [21,22]. These characteristics make RPA particularly suitable for on-site rapid detection.

The CRISPR-Cas system, based on Clustered Regularly Interspaced Short Palindromic Repeats (CRISPR) and CRISPR-associated proteins (Cas), has emerged as a next-generation platform for HAB monitoring [23,24,25]. Cas12a protein (Cpf1) specifically recognizes double-stranded DNA (dsDNA) containing PAM sequences (5′-TTTN-3′ or 5′-TTN-3′), activating RuvC domain-mediated cis-cleavage of target dsDNA and trans-cleavage of single-stranded DNA (ssDNA) [26]. This mechanism enables highly sensitive HAB detection using fluorescence-labeled or biotin-FAM-modified ssDNA reporters. Coupled with lateral flow dipstick (LFD) technology, visual readout is achieved within 1–2 min via carboxyfluorescein-biotin hybridization, offering instrument-free operation and significantly improved usability over traditional gel electrophoresis [27,28,29]. Currently, the RPA-CRISPR/LbaCas12a-LFD technology has been applied in clinical and microbiology diagnostics due to its rapidity, high specificity, and minimal equipment requirements; its application in HAB detection remains in the early stages [23,30,31]. In 2023, [32] first successfully applied this technology to detect *Karenia mikimotoi* (Dinoflagellata), demonstrating its feasibility for algal detection. Subsequently, [33] further extended this technology to *Prymnesium parvum* (Haptophyta) detection. In that study, the additions of CRISPR made the method more specific and 10,000 times more sensitive than standalone RPA detection of *P. parvum*. These advantages make this approach suitable for early warning detection and prevention of HAB events in environmental water. However, no studies have yet reported its application for detecting *C. dentata*.

This study developed a simple, highly sensitive, and specific rapid detection method for *C. dentata* by integrating RPA, CRISPR/LbaCas12a, and LFD technologies. Since detection accuracy and sensitivity are crucial for early monitoring, we focused on optimizing reaction conditions to improve the specificity and sensitivity of the RPA-CRISPR/LbaCas12a-LFD technique for targeting *C. dentata*. The method was validated using both spiked samples and field samples from the Bohai Sea, demonstrating strong applicability in real-world scenarios. This innovative approach effectively addresses the critical challenge of low detection rates for low-abundance algal cells during early HAB monitoring. Notably, it represents the first successful implementation of RPA-CRISPR-LbaCas12a-LFD technology for *C. dentata* detection. The developed method provides a reliable tool for rapid on-site early warning of harmful algal blooms, offering significant potential for marine ecosystem protection and bloom prevention.

## 2. Materials and Methods

### 2.1. Algal Species Culturing

In this study, ten algal species were chosen to evaluate the detection specificity of *Chrysotila dentata* (NMBjih026-1) with the RPA-CRISPR/Cas12a-LFD method based on their potential for interference. The selected species included *Prymnesium parvum* (NMBjih029), a coccolithophore within the Haptophyta phylum that shares genomic similarities with *C. dentata*; toxin-producing species such as *Karlodinium veneficum* (NMBjah047-1) and *Pseudo-nitzschia multiseries* (NMBguh002-1-1); and common bloom-forming species including *Rhodomonas salina* (NMByih012-1), *Thalassiosira pseudonana* (NMBguh006), *Skeletonema costatum* (NMBguh0042), *Karenia mikimotoi* (NMBjah052), *Heterosigma akashiwo* (NMBjah045), *Chaetoceros curvisetus* (NMBguh003-10), and *Phaeodactylum tricornutum* (CCMP2561). All these algal species potentially co-occur with *C. dentata* in natural water bodies and may consequently interfere with its detection to varying degrees. The algal strains *P. parvum*, *R. salina*, *T. pseudonana*, *S. costatum*, *K. mikimotoi*, *P. multiseries*, *K. veneficum*, *H. akashiwo*, and *C. curvisetus* were all provided by the Microalgae Culture Collection of Ningbo University. In addition, the strain *P. tricornutum* was provided by Westlake University, and *C. dentata* was supplied by Shanghai Jiao Tong University. All algal strains were cultured under the following conditions: f/2 medium, temperature maintained at 16 °C, light intensity of 15–20 μmol photons m^−2^ s^−1^ with a 12 h:12 h light–dark cycle. Cultures were maintained statically with manual shaking performed 1–2 times daily.

### 2.2. Construction of RPA-CRISPR/LbaCas12a-LFD System

**Figure 1:**
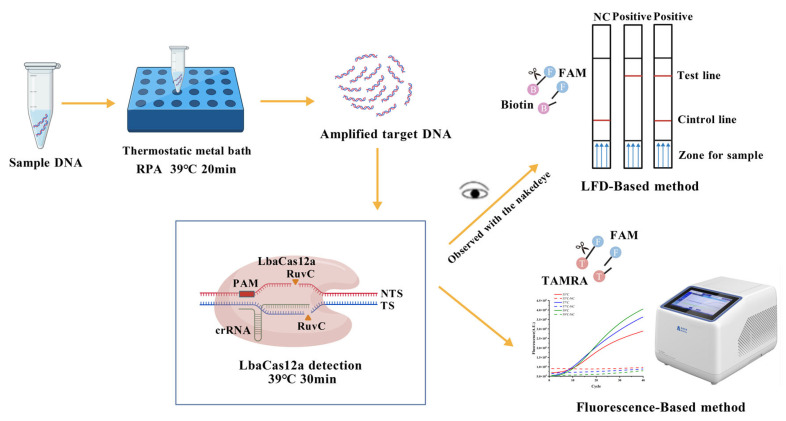
Working principle of the RPA-CRISPR/LbaCas12a-LFD system. Created with BioGDP.com (https://biogdp.com, accessed on 14 September 2025) [34].

This study employed RPA technology to amplify the ITS target sequence of *C. dentata*. The amplified products were then hybridized with crRNA to activate the trans-cleavage activity of Cas12a on ssDNA. To monitor the cleavage of ssDNA probes, we utilized two detection methods: fluorescence-based quantitative analysis and LFD detection (Figure 1). The fluorescence quantification method was primarily used to optimize RPA primers and determine the optimal CRISPR reaction temperature. However, considering that fluorescence detection requires specialized instrumentation, we subsequently adopted LFD for the final detection step.

The RPA reaction was performed using a DNA Isothermal Rapid Amplification Kit (Amplification Future, Changzhou, China) with a total reaction volume of 50 μL. The reaction mixture contained 29.5 μL rehydration buffer, 12 μL ddH_2_O, 2 μL each of forward and reverse primers (10 μM), 2 μL DNA template, and 2.5 μL MgOAc (280 mM). After thorough mixing, the reaction was incubated at 39 °C for 20 min in a metal bath (Allsheng, Hangzhou, China) to obtain RPA products. For the LFD-based CRISPR/Cas12a detection system, a 20 μL reaction was prepared containing 13.5 μL ddH_2_O, 0.5 μL LbaCas12a protein (2 μM, MAGIGEN, Guangzhou, China), 1 μL ssDNA-FB reporter (10 μM, 5′-FAM-TTATT-Biotin-3′, GENERAL BIOL, Chuzhou, China), 1 μL crRNA (50 nM), 2 μL 10× reaction buffer, and 2 μL RPA product. The mixture was incubated at 37 °C for 20 min in a dry bath incubator. Following the reaction, 4 μL of the product was transferred to a 96-well plate and mixed with 46 μL ddH_2_O. Finally, the sample loading zone of the LFD strip was immersed into the mixed solution and incubated for 2 min before result interpretation.

### 2.3. Design and Screening of crRNA and RPA Primers

The ITS sequence of *C. dentata* was selected as the target for crRNA design. A PAM site (TTT + N) was identified, and due to the low sequence specificity near the PAM region, only one target sequence was chosen for crRNA construction (5′-UAAUUUCUACUAAGUGUAGAUCGCUGCGCGUUGCGAGAGAUGCCG-3′). Two single-stranded crDNA oligonucleotides (crDNA-F: TAATACGACTCACTATAGGGTAATTTCTACTAAGTGTAGATCGCTGCGCGTTGCGAGAGATGCCG; crDNAR: CGGCATCTCTCGCAACGCGCAGCATCTACACTTAGTAGAAATTACCCTATAGTGAGTCGTATTA) were synthesized by General Biosystems (Chuzhou, China). The double-stranded crDNA was obtained by annealing the single-stranded crDNA fragments in a reaction mixture containing 13.8 μL Tris-HCl (10 mM), 2 μL NaCl (75 mM), 0.2 μL MgCl_2_ (1 mM), and 2 μL each of crDNA-F and crDNA-R. The annealing protocol consisted of an initial denaturation at 95 °C for 3 min, followed by 140 cycles with a gradual temperature decrease of 0.5 °C per cycle. Finally, the crRNA was transcribed in vitro using the T7 High Yield RNA Transcription Kit (Vacyme, Nanjing, China).

Based on the designed crRNA, forward and reverse RPA primers were designed at its flanking regions. To ensure primer specificity, the 28S rRNA sequence of *C. dentata* was aligned with closely related species, including *Helladosphaera* sp. (EU729465.1), *Hymenomonas coronata* (EU729466.1), and *Calyptrosphaera sphaeroidea* (EU729466.1), confirming that the primers would not cross-react with non-target sequences. Following the design guidelines of the TwistAmp^®^ DNA Amplification Kit (TwistDX, Cambridge, UK), three RPA primer pairs were synthesized. The amplification efficiency of these primers was first evaluated by performing RPA reactions, followed by 2% agarose gel electrophoresis to verify product formation. The three RPA primer pairs were subsequently screened using a fluorescence-based detection method. The 20 μL reaction mixture consisted of 13.5 μL ddH_2_O, 0.5 μL LbaCas12a protein (2 μM), 1 μL ssDNA-FB reporter (10 μM, 5′-FAM-TTATT-TAMRA-3′), 1 μL crRNA (50 nM), 2 μL 10× reaction buffer, and 2 μL RPA product. After preparing the reaction on ice, CRISPR-mediated cleavage was performed using an Applied Biosystems™ QuantStudio™ 1 real-time PCR instrument (Thermo Fisher Scientific, Carlsbad, CA, USA). Fluorescence signals were monitored under the following conditions: 37 °C for 20 min, with readings taken every 30 s. The primer pair yielding the highest fluorescence signal was selected for downstream experiments.

### 2.4. Optimization of RPA-CRISPR/LbaCas12a-LFD Reaction System

#### 2.4.1. Optimization of ssDNA Reporter Concentration

The optimization of ssDNA concentration was performed using the LFD-based detection method. Four concentration gradients of ssDNA were tested: 500 nmol L^−1^, 50 nmol L^−1^, 5 nmol L^−1^, and 0 nmol L^−1^. The optimization reaction system consisted of 1 μL ssDNA reporter and 19 μL ddH_2_O. The optimal ssDNA concentration was determined by selecting the test strip showing the faintest detection line color, which was then used for subsequent experiments.

#### 2.4.2. Optimization of Reaction Time

Based on the optimized ssDNA concentration determined previously, we further screened the reaction time using the LFD method with four gradients (10 min, 20 min, 30 min, and 40 min). The optimal reaction time was selected according to the LFD strip results, specifically choosing the time point that showed the darkest test line color and the lightest control line color for subsequent experiments.

#### 2.4.3. Optimization of Reaction Temperature

To optimize the CRISPR reaction temperature and improve detection sensitivity for positive results, we conducted temperature screening based on the predetermined optimal ssDNA concentration and reaction time. Using a fluorescence-based detection method, three temperature gradients (35 °C, 37 °C, and 39 °C) were tested. The optimal reaction temperature was selected according to the highest fluorescence values obtained from the detection system.

### 2.5. Specificity and Sensitivity Evaluation

The specificity assessment was conducted using the optimized RPA-CRISPR/LbaCas12a-LFD reaction system, with genomic DNA from 11 algal species (Section 2.1) serving as templates for specificity testing, while ddH_2_O was used as the negative control template. To evaluate the method’s sensitivity, genomic DNA extracted from log-phase *C. dentata* cultures (5 × 10^4^ pg μL^−1^) was serially diluted with ddH_2_O to create a concentration gradient (ranging from 5 × 10^2^ pg μL^−1^ to 5 × 10^−8^ pg μL^−1^). Each diluted sample was then analyzed using the optimized RPA-CRISPR/LbaCas12a-LFD detection system.

### 2.6. Environmental Water Samples Analysis

To verify the practical applicability of this method, we evaluated its performance using both spiked samples and field sample seawater. Seawater was collected from the East China Sea between July and August 2024 and filtered through 0.22 μm polycarbonate membranes. After microscopic examination confirmed the absence of *C. dentata*, known quantities of *C. dentata* cells were introduced to generate simulated samples with eight distinct concentration gradients (2.82 × 10^4^ cells mL^−1^, 2.82 × 10^3^ cells mL^−1^, 2.82 × 10^2^ cells mL^−1^, 2.82 × 10^1^ cells mL^−1^, 2.82 × 10^0^ cells mL^−1^, 2.82 × 10^−1^ cells mL^−1^, 2.82 × 10^−2^ cells mL^−1^, and 2.82 × 10^−3^ cells mL^−1^). For field validation, seawater samples were collected from 10 monitoring stations in the Bohai Sea. Field samples (2 L surface water) were pre-filtered through 200 μm mesh to remove large suspended particles, zooplankton, and phytoplankton, followed by secondary filtration through 0.22 μm polycarbonate membranes (Millipore, Burlington, MA, USA) using a vacuum filtration pump (<50 kPa). The filter membranes were transferred in tubes and were then snap-frozen in liquid nitrogen and stored at −80 °C. Subsequently, genomic DNA was extracted from filter membrane samples (Magen, Guangzhou, China) and served as a template (2 μL) for RPA-CRISPR/LbaCas12a-LFD detection to validate field applicability.

## 3. Results

### 3.1. Optimal crRNA and RPA Primers

**Figure 2:**
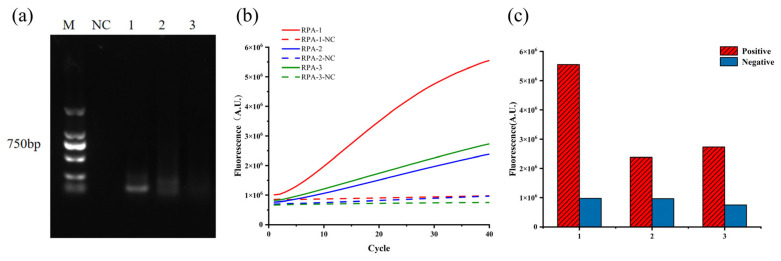
Screening of optimal RPA primers: (**a**) Agarose gel electrophoresis (2%) for optimal RPA primer selection; (**b**) Real-time fluorescence curves of RPA primer screening; (**c**) Comparison of maximum fluorescence values from RPA primer screening. Here, M: DNA marker; NC: negative control; 1: RPA-1; 2: RPA-2; 3: RPA-3. RPA-1-NC, RPA-2-NC, and RPA-3-NC represent their respective negative controls.

Based on the designed crRNA, 3 pairs of RPA primers (RPA-1, RPA-2, and RPA-3) were designed and screened. Analysis of the RPA products by 2% agarose gel electrophoresis revealed distinct bands for RPA-1 and RPA-2, while RPA-3 showed only faint bands (Figure 2a), indicating higher amplification efficiency for RPA-1 and RPA-2 compared to RPA-3. CRISPR fluorescence detection demonstrated that all three primer pairs produced negative control signals below 1,000,000 A.U. (Figure 2c), meeting the established negative criteria. Notably, RPA-1 exhibited significantly higher positive fluorescence values than RPA-2 and RPA-3 (Figure 2b). Based on these results, RPA-1 was selected as the optimal primer pair for subsequent experiments.

### 3.2. Optimizing the Reaction Conditions for the RPA-CRISPR/LbaCas12a-LFD Platform

#### 3.2.1. Optimal ssDNA Reporter Concentration

**Figure 3:**
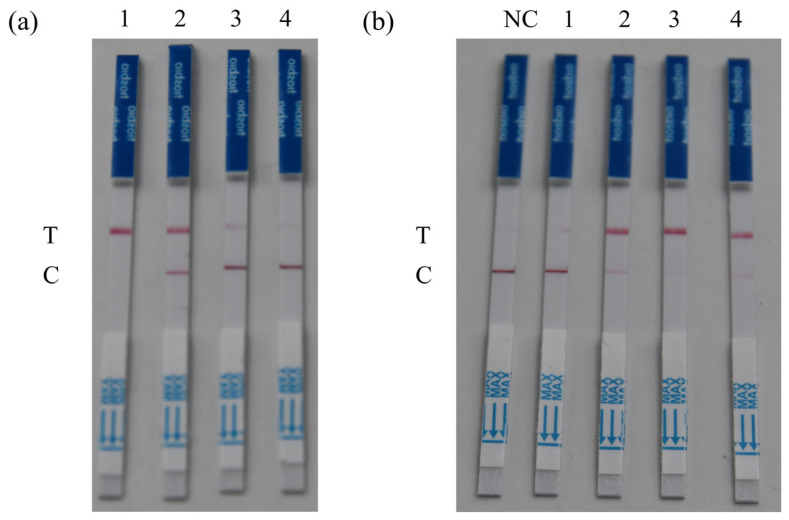
Optimization of CRISPR/LbaCas-LFD reaction conditions: (**a**) ssDNA concentration optimization (negative controls). Here, 1: 0 nmol L^−1^; 2: 5 nmol L^−1^; 3: 50 nmol L^−1^; 4: 500 nmol L^−1^; (**b**) CRISPR/Cas reaction time optimization (positive results). Here, 1: 10 min; 2: 20 min; 3: 30 min; 4: 40 min; T: the test line; C: the control line.

Four concentration gradients of ssDNA reporter were tested to determine the optimal concentration. The results demonstrated that within the range of 0–500 nmol L^−1^, the color intensity of the negative test line decreased progressively with increasing ssDNA reporter concentration. At 500 nmol L^−1^, the test line appeared faintest and was nearly invisible (Figure 3a). When negative results showed the faintest test line or no visible test line coloration, this indicated minimal probability of false positives and highest detection reliability. Consequently, 500 nmol L^−1^ was ultimately selected as the optimal ssDNA concentration for follow-up experiments.

#### 3.2.2. Optimal Reaction Time

To enhance detection sensitivity while maintaining reaction efficiency, we optimized the CRISPR incubation time using the established optimal ssDNA concentration. The results showed that among the tested reaction times from 10 to 40 min, the lateral flow strips exhibited the darkest test line color at 30 min (Figure 3b), and thus 30 min was chosen as the optimal CRISPR reaction time for the following experiments.

#### 3.2.3. Optimal Reaction Temperature

**Figure 4:**
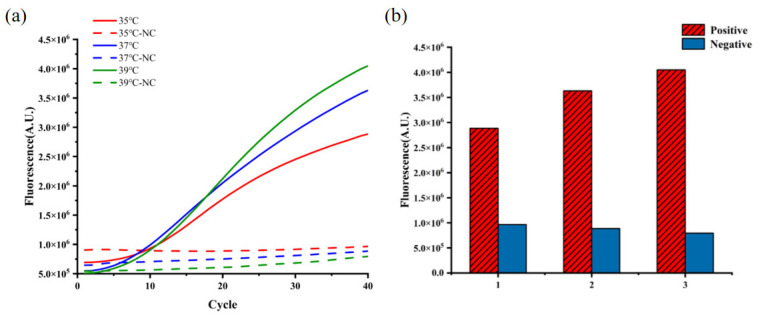
Optimization of CRISPR/LbaCas-LFD reaction temperature: (**a**) Real-time fluorescence curves of temperature screening; (**b**) Comparison of maximum fluorescence values across tested temperatures. Here, NC represents the negative control. 1: 35 °C; 2: 37 °C; 3: 39 °C.

To optimize the temperature conditions for CRISPR reactions, we conducted temperature screening based on the optimal ssDNA concentration and reaction time. The results demonstrated that within the tested range of 35 °C to 39 °C, the fluorescence intensity of CRISPR reactions increased progressively with rising temperature. At 39 °C, the reaction demonstrated the highest positive fluorescence peak compared to other tested temperatures, along with the lowest negative control fluorescence peak that remained below 1,000,000 A.U. (Figure 4), successfully meeting predefined negative control requirements. Based on these results, 39 °C was selected as the optimal reaction temperature and implemented in all subsequent experimental procedures.

### 3.3. Evaluation of Detection Specificity and Sensitivity

**Figure 5:**
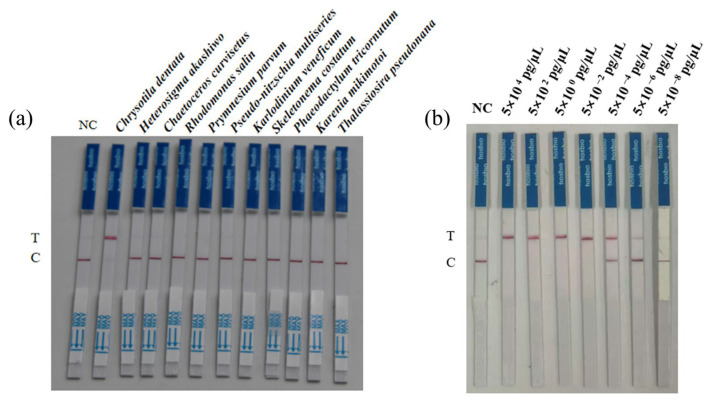
Specificity and sensitivity evaluation of the RPA-CRISPR/LbaCas12a-LFD method for *C. dentata* detection: (**a**) Specificity validation of *C. dentata* detection; (**b**) Sensitivity assessment of *C. dentata* detection. Here, NC: negative control; T: the test line; C: the control line.

To evaluate the specificity of our newly developed RPA-CRISPR/LbaCas12a-LFD detection method for *C. dentata*, we selected 10 potential interfering algal species for validation testing. All these algal species potentially co-occur with *C. dentata* in natural water bodies and may consequently interfere with its detection to varying degrees. The results showed that only *C. dentata* produced a visible test line on the LFD strips, while all other species showed no coloration (Figure 5a), demonstrating the method’s high specificity for *C. dentata* detection without cross-reactivity or false positives with these potential interfering species.

Sensitivity serves as a critical parameter for evaluating detection methods, with higher sensitivity corresponding to improved detection performance and enhanced early warning capability, thereby indicating greater methodological reliability. Results demonstrated that the optimized RPA-CRISPR/LbaCas12a-LFD assay achieved a remarkable sensitivity of 5 × 10^−6^ pg μL^−1^ (Figure 5b).

### 3.4. Testing of Environmental Water Samples

**Figure 6:**
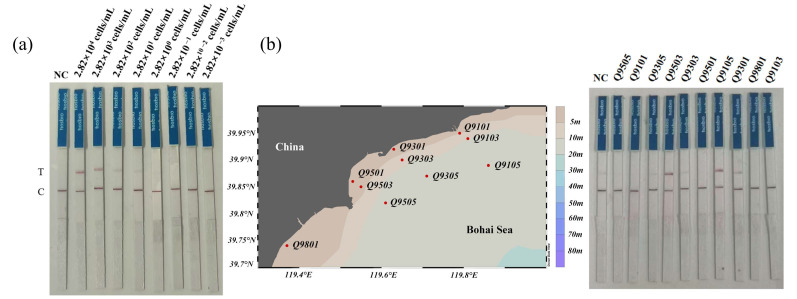
Validation of the RPA-CRISPR/LbaCas12a-LFD method for *C. dentata* detection using simulated and field-collected samples: (**a**) Performance evaluation with simulated field samples; (**b**) Application to authentic field samples from the Bohai Sea. Here, NC: negative control.

To evaluate potential interference from environmental water samples on the RPA-CRISPR/LbaCas12a-LFD detection of *C. dentata*, we performed simulated field tests using spiked samples. The results showed that the RPA-CRISPR/LbaCas12a-LFD method remained effective for *C. dentata* detection in spiked samples, achieving a high sensitivity of 2.82 × 10^1^ cells mL^−1^ (Figure 6a).

To validate the feasibility of the RPA-CRISPR/LbaCas12a-LFD method for detecting *C. dentata* in field environments, we collected and tested 10 natural water samples from the Bohai Sea. The detection results showed that among these field samples, 3 tested positive—including 1 weakly positive and 2 strongly positive samples (Figure 6b). These results demonstrate that the method is suitable for field water sample detection and can be further applied to field monitoring. More importantly, they not only reveal the widespread distribution of *C. dentata* in Bohai Sea coastal waters that warrants serious attention but also confirm the necessity of developing rapid field detection methods for this species.

## 4. Discussion

HABs have emerged as a critical environmental issue in China, posing substantial threats to marine ecosystem functioning, aquaculture industries, and public health [35,36]. Coccolithophores, in particular, may compromise ecosystem stability during bloom events due to their calcification characteristics. Although the organic matter and calcified products from such blooms can supply nutrients for zooplankton and microbial communities, the decay of large-scale blooms may induce ecological risks such as localized hypoxia and alterations in food web structure [37]. Consequently, routine monitoring of harmful algal species carries significant ecological importance.

This study developed a novel RPA-CRISPR/LbaCas12a-LFD detection method for *C. dentata*, leveraging the CRISPR/Cas system to enable efficient field detection. The technology integrates three key modules, including RPA isothermal amplification, CRISPR/Cas12a specific recognition, and LFD visual detection, establishing a complete “amplification-identification-visualization” detection system [32,33]. The RPA technology utilizes recombinase-mediated isothermal amplification to overcome the dependence on thermal cyclers required by conventional PCR [38], while the CRISPR/LbaCas12a system ensures specificity through PAM site recognition and trans-cleavage activity. Results are visually interpretable within 1–2 min using LFD strips. This innovative approach not only offers simple operation and rapid reaction characteristics but also breaks through instrumentation limitations for field detection, providing reliable technical support for in situ monitoring of harmful algal blooms.

Despite its considerable advantages, this detection method faces critical challenges in practical applications, particularly regarding the design of specific primers and optimization of reaction conditions. High-specificity primer design requires careful consideration of both target sequence selection and PAM site constraints, while system optimization necessitates a balance among sensitivity, specificity, and environmental adaptability. To address these technical difficulties, this study focused on resolving key issues, including species-specific primer screening and reaction condition improvement.

The effective cleavage function of the CRISPR system is highly dependent on both the presence of PAM sites and the specific design of crRNA, which somewhat limits primer selection options. Different crRNA designs and PAM sites may also exhibit varying cleavage activities [39,40,41]. In this study, analysis of *C. dentata* 18S rRNA, 28S rRNA, and ITS sequences yielded only one target sequence that simultaneously met both PAM site and crRNA design requirements. This suggests potential limitations when extending this method to detect other algal species that may lack suitable PAM sites in their target sequences. Recent studies, however, have shown that Cas12a protein activity can still be effectively triggered with a simplified PAM motif (e.g., TTN), which may reduce crRNA design challenges [42]. For toxin-producing algae, targeting toxin synthesis-related genes for primer design offers an alternative approach. It should be noted that RPA technology carries potential aerosol contamination risks, prompting researchers to develop closed-tube reaction methods to minimize contamination and false results [43,44]. These potential improvements could facilitate broader application of CRISPR-based detection in algal monitoring.

The RPA-CRISPR/LbaCas12a-LFD detection system developed in this study demonstrates high specificity and sensitivity, outperforming both standalone RPA and CRISPR-based methods [45,46].

Moreover, in contrast to traditional microscopy, the technique incorporates optimized reaction conditions, including probe temperature and incubation time, thereby improving its applicability for monitoring harmful algal blooms (HABs) in marine settings. The entire detection process is completed within one hour, significantly shortening the time required for HAB detection while eliminating the need for sophisticated instrumentation, underscoring its efficiency compared to existing approaches. Despite these strengths, the current method still requires improvements in areas such as primer design limitations and the control of aerosol contamination. Future developments could focus on designing primers that target genes associated with toxin synthesis to improve detection accuracy for a wider range of toxic microalgae [47]. Furthermore, adopting closed-tube reaction systems may help minimize aerosol contamination and decrease operational errors. In summary, the RPA-CRISPR/LbaCas12a-LFD detection method established in this study offers a reliable technical platform for the field-based detection of HABs.

## 5. Conclusions

This study successfully established a rapid detection method for *C. dentata* based on RPA-CRISPR/LbaCas12a-LFD technology, providing a novel technical approach for harmful algal monitoring. The assay integrates RPA isothermal amplification with CRISPR/Cas12a-specific recognition and lateral flow dipstick (LFD) visualization, eliminating the need for sophisticated instruments while significantly enhancing detection efficiency and operational simplicity. The complete detection process can be accomplished within 1 h, exhibits high specificity for *C. dentata* by effectively distinguishing it from 10 non-target algal species, and achieves a detection sensitivity of 5 × 10^−6^ pg μL^−1^. These features make the RPA-CRISPR/LbaCas12a-LFD technology particularly suitable for field monitoring of HAB species, supporting early warning and preventive management through rapid on-site detection.

## Data Availability

The original contributions presented in the study are included in the article, further inquiries can be directed to the corresponding authors.
